# Biosynthesis of Nutraceutical Fatty Acids by the Oleaginous Marine Microalgae *Phaeodactylum tricornutum* Utilizing Hydrolysates from Organosolv-Pretreated Birch and Spruce Biomass

**DOI:** 10.3390/md17020119

**Published:** 2019-02-15

**Authors:** Alok Patel, Leonidas Matsakas, Kateřina Hrůzová, Ulrika Rova, Paul Christakopoulos

**Affiliations:** Biochemical Process Engineering, Division of Chemical Engineering, Department of Civil, Environmental and Natural Resources Engineering, Luleå University of Technology, 971-87 Luleå, Sweden; alok.kumar.patel@ltu.se (A.P.); katerina.hruzova@ltu.se (K.H.); ulrika.rova@ltu.se (U.R.); paul.christakopoulos@ltu.se (P.C.)

**Keywords:** polyunsaturated fatty acids, EPA, DHA, marine algae, *Phaeodactylum tricornutum*, forest biomass

## Abstract

Polyunsaturated fatty acids (PUFAs) are essential for human function, however they have to be provided through the diet. As their production from fish oil is environmentally unsustainable, there is demand for new sources of PUFAs. The aim of the present work was to establish the microalgal platform to produce nutraceutical-value PUFAs from forest biomass. To this end, the growth of *Phaeodactylum tricornutum* on birch and spruce hydrolysates was compared to autotrophic cultivation and glucose synthetic media. Total lipid generated by *P. tricornutum* grown mixotrophically on glucose, birch, and spruce hydrolysates was 1.21, 1.26, and 1.29 g/L, respectively. The highest eicosapentaenoic acid (EPA) production (256 mg/L) and productivity (19.69 mg/L/d) were observed on spruce hydrolysates. These values were considerably higher than those obtained from the cultivation without glucose (79.80 mg/L and 6.14 mg/L/d, respectively) and also from the photoautotrophic cultivation (26.86 mg/L and 2.44 mg/L/d, respectively). To the best of our knowledge, this is the first report describing the use of forest biomass as raw material for EPA and docosapentaenoic acid (DHA) production.

## 1. Introduction

Most naturally occurring fatty acids have an unbranched chain consisting of an even number of carbon atoms ranging from 4 to 28. Depending on the nature of the hydrocarbonated chain, the fatty acids can be saturated, monounsaturated, or polyunsaturated [1]. Many fatty acids can be synthesized by humans, however not some polyunsaturated fatty acids (PUFAs), such as omega-3 (n−3) and omega-6 (n−6) fatty acids [2]. The parent constituents of omega-3 and omega-6 fatty acids are α-linolenic acid (C_18:3_ n−3, ALA) and linoleic acid (C_18:2_ n−6, LA), respectively. Omega-6 fatty acids, such as arachidonic acid (C_20:4_ n−6; AA), can be synthesized by humans from LA, whereas the essential omega-3 fatty acids, such as eicosapentaenoic acid (C_20:5_ n−3; EPA), docosapentaenoic acid (C_22:5_ n−3, DPA), and docosahexaenoic acid (C_22:6_ n−3, DHA), can be synthesized from ALA. However, the conversion rate of ALA to EPA, DPA, and DHA is very low [3]. Consequently, both omega-6 and omega-3 PUFAs have to be taken up through the diet, preferably at a ratio of 5:1 or less [4]. Presently, the most common source of omega-3 PUFAs is represented by fish of the Salmonidae, Scombridae, and Clupeidae families which contain a high percentage of DHA and EPA [3]. 

The ever-rising global demand for omega-3 PUFAs cannot be met by fish oil due to diminishing fish stocks and pollution of marine ecosystems [5] which has led to increased interest in alternative sustainable sources. Vegetable oils from genetically engineered plant oilseeds and microorganisms are two potential alternatives to fish oil, even though omega-3 PUFAs are highest in the latter. *Brassica juncea*, *Arabidopsis thaliana*, and *Camelina sativa* are some examples of genetically engineered plant species with potential to accumulate omega-3 PUFAs [6]. Although transgenic plants present numerous advantages, their production is dependent on seasonal and climatic conditions and the availability of arable land. Moreover, there are public concerns regarding the cultivation of transgenic crops in open ecosystems. These, together with regulatory issues, restrict the large-scale production of genetically modified crops [7]. Microorganisms are known natural producers of microbial oils similar to those obtained from plants and animals and a possible source of nutritionally important omega-3 PUFAs [8]. The use of microorganisms benefits from the ability to use biochemical engineering to improve microbial growth rates, low nutrient requirement to achieve growth, easily controllable culture conditions, and availability of well-annotated genomes and metabolic pathways that allow their genetic manipulation [9]. Moreover, microbial oils usually contain a significant amount of natural antioxidants, such as carotenoids and tocopherols, which play an important role in protecting omega-3 PUFAs from oxidation and therefore improve their storage stability. The first commercial product obtained from microbial oil was a gamma-linolenic acid (C_18:3_ n-6)-rich oil produced using the filamentous fungus *Mucor circinelloides* and its production lasted from 1985 until 1990 [10].

Oleaginous microalgae constitute microscopic bio-factories that are capable of producing elevated amounts of oil which can be used as feedstock for omega-3 PUFAs [11]. A number of algal species, such as *Nitzschia*, *Navicula*, *Nannochloropsis*, *Phaeodactylum*, and *Porphyridium*, have been identified as producers of omega-3 PUFAs [6,12]. The utilization of oleaginous microalgae for microbial oil production has many advantages over other non-conventional sources; they attain higher lipid productivity than plants, allow for easy scale-up of upstream and downstream processing, and are less influenced by seasonal or climatic variations. In addition, oleaginous microalgae can utilize numerous low-cost renewable substrates for growth and lipid accumulation [13]. Finally, as opposed to fungi, which form mycelia, microalgae are made of single cells, facilitating their handling in large-scale cultivations [14]. Diatoms are one of the most productive classes of microalgae and can easily adapt to environmental changes [15]. Almost all diatom species can grow photoautotrophically, with a few of them able to grow heterotrophically. *Phaeodactylum tricornutum* utilizes glucose in the presence of light and glucose makes up to 90% of the carbon assimilated into biomass under exponential growth [16]. Transcriptome analysis revealed no correlation between the expression of membrane glucose transporters and light or glucose exposure. Hence, the inability of *P. tricornutum* to grow on glucose in the dark was attributed to the low expression of glucose transporters [16]. As a result, *P. tricornutum* represents a facultative mixotroph that cannot grow heterotrophically on organic carbon in the absence of light. More recently, *P. tricornutum* was shown to grow heterotrophically in the dark on various organic carbon sources following the engineered introduction of glucose transporters [17]. The production of biomass and lipids from microalgae is strongly affected by the mode of cultivation as microalgae can be cultivated photoautotrophically, mixotrophically, heterotrophically, and photoheterotrophically [18]. Heterotrophic and mixotrophic conditions are considered advantageous over photoautotrophic cultivation due to the higher productivity, lipid concentration, and lipid content that can be achieved [19]. However, the high cost associated with organic carbon sources is a major bottleneck for the commercialization of the above process. The use of non-edible lignocellulosic materials and industrial waste as sources of sugars could reduce overall production costs, thereby aiding the transition to large-scale lipid production [13]. Among the various alternatives, wood biomass is an excellent option for countries such as Sweden with as high as 53.1% of its land covered by forests, a thriving forest-based industry, and sustainable forest management [20]. Norway spruce (*Picea abies*) and birch (*Betula pendula* and *Betula pubescens*) account for 40.8% and 12.4%, respectively, of the total standing volume of forest (3490 million m^3^sk) [20]. As these two tree species represent characteristic examples of hardwood and softwood, we developed a novel hybrid organosolv–steam explosion method that yields fractionated, pretreated solids with high cellulose content [21,22]. The ensuing hydrolysates were effectively utilized for the heterotrophic growth of *Auxenochlorella protothecoides* by our group, producing very high lipid yields [23]. Here, we intended to evaluate the use of these hydrolysates for the production of fatty acids of nutraceutical value as an alternative and novel approach. To this end, we developed a cost-effective process for the cultivation of *P. tricornutum*, a species capable of producing EPA-rich lipids, on forest hydrolysates. To the best of our knowledge, this is the first report of forest biomass being used for the production of nutraceutical fatty acids from microalgae.

## 2. Results and Discussion

### 2.1. Effect of Various Initial Glucose Concentrations on the Growth and Lipid Accumulation of P. tricornutum under Mixotrophic Cultivation

Photoautotrophic cultivation requires light as an energy source, CO_2_ as an inorganic carbon source, and nutrients to support microalgae growth [24]. However, some microalgae show enhanced growth under mixotrophic cultivation whereby photoautotrophic and heterotrophic metabolism are simultaneously active, allowing the use of organic carbon sources and, consequently, higher lipid and biomass productivity [25]. To compare the effect of photoautotrophic versus mixotrophic cultivation on biomass and lipid accumulation by *P. tricornutum*, different initial levels of glucose ranging from 0 g/L (control) to 10 g/L were added to basal F/2 medium with yeast extracted as an organic nitrogen source, while the photoautotrophic cultivation was performed with sodium nitrate as an inorganic nitrogen source instead of yeast extract. The C/N ratio was adjusted to 20 and each tube was inoculated with a 10% volume of exponentially growing seed culture. The results for cell dry weight, biomass yield, total lipid concentration, lipid yield, and lipid content of *P. tricornutum* grown on various initial concentrations of glucose are presented in Table 1. The highest cell dry weight (4.32 ± 0.32 g/L) and biomass productivity (0.332 ± 0.009 g/L/d) along with total lipid concentration (1.16 ± 0.23 g/L) and lipid productivity (0.089 ± 0.002 g/L/d) were observed when *P. tricornutum* was supplemented with 10 g/L glucose. Overall, biomass concentration increased from 3.38 ± 0.16 g/L to 4.32 ± 0.32 g/L and biomass productivity increased from 0.260 ± 0.003 g/L/d to 0.332 ± 0.009 g/L/d when microalgae were shifted from 2 g/L to 10 g/L glucose (Table 1). However, biomass yield based on glucose consumption followed an opposite trend, decreasing from 1.69 ± 0.19 g/g_substrate_ (2 g/L glucose) to 1.34 ± 0.19 g/g_substrate_ (10 g/L glucose). Moreover, an increase in glucose concentration above 2 g/L resulted in a significant portion of it (up to 68% of the initial input) to remain untouched (Table 2). Lipid concentration increased with increasing glucose concentration from 0.88 g/L (2 g/L glucose) to 1.08 g/L (4 g/L glucose), while lipid yield was significantly reduced from 0.44 ± 0.09 g/g_substrate_ to 0.34 ± 0.08, respectively; beyond 4 g/L of glucose, any further increase had only a minor impact on lipid concentration (Table 1). The lower biomass and lipid yields, together with the unconsumed glucose had a negative impact on the economics of the process and thus the addition of 2 g/L was chosen for further experiments. The addition of even this small amount of glucose increased the cell dry weight and lipid concentration by approximately 1.34 and 1.54 times, respectively, compared to the culture without any sugar source (glucose). The result obtained with inorganic nitrogen in the photoautotrophic cultivation showed a lower amount of cell dry weight (0.89 ± 0.11 g/L) and lipid concentration (0.20 ± 0.06 g/L) than those obtained with yeast extract as an organic nitrogen source in mixotrophic cultivation without glucose (Table 1). Mixotrophic growth of the obligate photoautotrophic microalga *P. tricornutum* in the presence of various organic carbon sources has been described previously [15,19,26]. Liu et al. (2009) reported that when *P. tricornutum* was cultivated photoautotrophically, maximum biomass was 460 ± 3 mg/L which was increased to 555 ± 10 mg/L, 587 ± 8 mg/L, and 713 ± 11 mg/L under mixotrophic growth on 100 mM glucose, acetate, and glycerol, respectively, however, the cultivation conditions were different from this study [19]. In another study, when *P. tricornutum* was cultivated on the soluble fraction of raw potato under a semi-continuous mode, cell productivity was 2.4 times higher than with photoautotrophic cultivation [27].

### 2.2. Effect of Various C/N Ratios on the Growth and Lipid Accumulation of P. tricornutum

**Figure 1:**
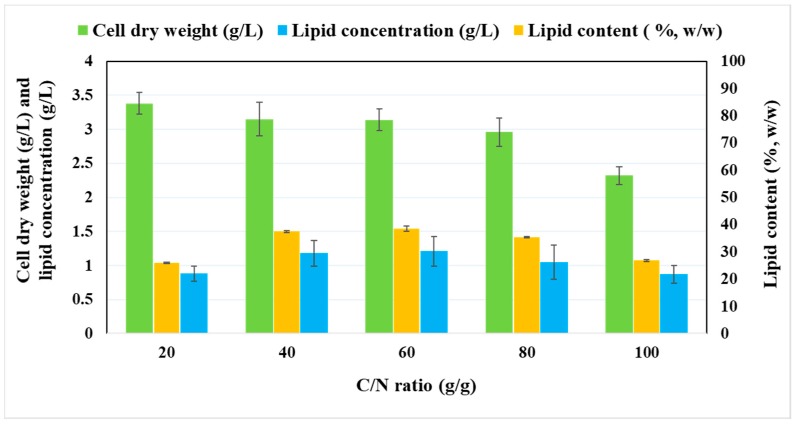
Effect of various C/N ratios on biomass and lipid production in *P. tricornutum* after 312 h of culture.

Lipid synthesis in oleaginous microorganisms depends on cultivation conditions and, most prominently, on the metabolic accessibility of nutrients. These microorganisms can synthesize elevated quantities of lipids under different stress conditions, including abiotic stress [28]. The most common abiotic stress is deficiency of a key nutrient together with excess amounts of carbon, which then triggers lipid accumulation pathways [29]. In general, lipid accumulation is significantly affected by the quality and quantity of key nutrients such as carbon, nitrogen, phosphorus, and sulfur as well as their ratios (C/N, C/P, C/S) [29]. Accordingly, after optimizing the initial concentration of glucose in the medium for maximum biomass yield (g/g), the lipid concentration was optimized by varying the concentration of nitrogen and subsequently the C/N ratio (Figure 1). An increase in the C/N ratio had a negative impact on the cell dry weight, which decreased from 3.38 ± 0.16 g/L (C/N 20) to 2.32 ± 0.13 g/L (C/N 100) (Figure 1), while the lipid concentration increased from 0.88 ± 0.11 g/L (C/N 20) to 1.21 ± 0.22 g/L (C/N 60). However, any further increment in the C/N ratio resulted in a decrease in lipid concentration (Figure 1). Given that the highest lipid content (38.53 ± 0.87% *w*/*w*) was attained at C/N 60, this ratio was selected as optimal for the subsequent batch cultivation of the algae using wood hydrolysates. An analogous positive effect of lower nitrogen concentration on lipid production was observed previously during photoautotrophic growth of *P. tricornutum* [30]. Nitrogen starvation is regarded as the best strategy to enhance lipid accumulation in oleaginous microorganisms [31]. Under nitrogen-limiting conditions, microalgae undergo rapid metabolic remodelling as nitrogen plays an important role in the synthesis of proteins, chlorophyll, and nucleic acids. However, the molecular synthesis of fatty acids differs from species to species and depends mainly on cultivation mode [30]. Diatoms are heterokont algae and are believed to have originated from secondary endosymbiotic events whereby a photoautotrophic alga was engulfed by a heterotrophic eukaryotic host [32]. Nitrogen uptake in diatoms is similar to that in other photoautotrophs. The assimilated nitrogen in the form of nitrate is reduced to nitrite by a cytosolic NADH-dependent nitrate reductase and transported to the chloroplast where it is reduced further to ammonium by a cyanobacterium-like ferredoxin-dependent nitrite reductase [33,34]. Ammonium is then assimilated into amino acids and other nitrogenous compounds by the joint action of plastid-localized glutamine synthetase (GSII) and glutamate synthase (GOGAT) [34]. In diatoms, carbon metabolism and nitrogen assimilation are linked via the tricarboxylic acid (TCA) cycle through 2-oxoglutarate and oxaloacetate [35]. Amino acid catabolism provides several intermediates for the TCA cycle which generates acetyl CoA which is used in fatty acid elongation [35].

### 2.3. Mixotrophic Cultivation of P. tricornutum on Wood Hydrolysates

*P. tricornutum* can assimilate organic carbon through mixotrophic cultivation [36]. The use of glucose derived from a renewable and non-edible resource such as lignocellulosic biomass can serve as an excellent way to promote low-cost commercial scale PUFA production. Here, we selected two sources of forest biomass to study the mixotrophic cultivation of *P. tricornutum* for EPA production, namely birch and spruce woodchips. To overcome the natural recalcitrance of lignocellulosic biomass, we improved the release of monomeric sugars by applying a pretreatment hybrid organosolv–steam explosion step [21,22] prior to enzymatic hydrolysis. As a result, 77.07 g/L and 64.70 g/L of glucose were produced after enzymatic hydrolysis of birch and spruce, corresponding to 89% and 80.9% cellulose hydrolysis [23], respectively. 

**Figure 2:**
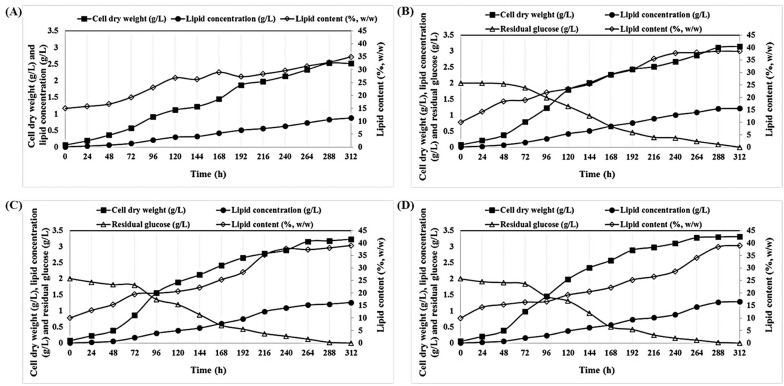
Time course of the growth and lipid accumulation of *P. tricornutum* cultivated under mixotrophic cultivation without using any sugar source (**A**) and mixotrophic cultivations using pure glucose, 2 g/L (GFM; C/N 60) (**B**), birch hydrolysate (BH; C/N 60) (**C**), and spruce hydrolysate (SH; C/N 60) (**D**).

Figure 2 shows a time-course of *P. tricornutum* grown mixotrophically using pure glucose (GFM; glucose, 2 g/L; C/N 60), birch (BH; glucose, 2 g/L; C/N 60), or spruce hydrolysate (SH; glucose, 2 g/L; C/N 60) and a comparison with the cultivation where no sugar source (i.e., glucose) was used. Cell dry weight, lipid concentration, and lipid content were 3.15 ± 0.53 g/L, 1.21 ± 0.19 g/L and 38.41 ± 0.21% *w*/*w*, respectively, when this alga was grown in pure glucose (GFM; glucose, 2 g/L; C/N 60) (see Table 2). These values were higher than those obtained with mixototrophic cultivation without any sugar source (glucose): 2.55 ± 0.24 g/L, 0.59 ± 0.25 g/L and 23.13 ± 0.32% *w*/*w*, respectively (Figure 2A). The highest cell dry weight and lipid concentrations were 3.31 ± 0.28 g/L and 1.29 ± 0.18 g/L, respectively, for spruce hydrolysate and 3.23 ± 0.32 g/L and 1.26 ± 0.11 g/L, respectively, for birch hydrolysate (Table 2). The results obtained with birch and spruce hydrolysates were similar to those attained with pure glucose, demonstrating the superiority of the hybrid organosolv–steam explosion method as it results in readily fermentable hydrolysates. The highest biomass (0.254 ± 0.007 g/L/d) and lipid (99.23 ± 1.09 mg/L/d) productivities were obtained when *P. tricornutum* was grown on spruce hydrolysate (Table 2).

### 2.4. EPA and DHA Production under Photoautotrophic and Mixotrophic Cultivation

*P. tricornutum* has substantial potential for the synthesis of long-chain omega-3 PUFAs of nutraceutical value and particularly EPA [37]. Generally, EPA content is dependent on the carbon source provided in mixotrophic culture [36]. The fatty acid profile of lipids extracted from *P. tricornutum* grown photoautotrophically and mixotrophically on glucose, birch, and spruce hydrolysates is presented in Table 3. Under photoautotrophic conditions, the fatty acid profile consists mainly of C_14:0_ (8.24%), C_16:0_ (15.39%), C_18:0_ (4.32%), C_16:1_ (17.23%), and C_18:1_ n9t (15.21%), with EPA (C_20:5_) and DHA (C_22:6_ n3) amounting to 13.43% and 1.65%, respectively (Table 3). The fatty acid profile was changed when the cultivation was carried out with yeast extract as organic nitrogen without using any sugar source under mixotrophic condition in which the main fatty acids were C_16:0_ (13.62%), C_16:1_ (13.62%), and C_18:1_ n9t (18.3%), with EPA (C_20:5_) and DHA (C_22:6_ n3) amounting to 14.0% and 1.71%, respectively. A switch of cultivation at C/N 20 with glucose (2 g/L) had a positive impact on EPA and DHA content which increased to 16.76% and 2.82%, respectively (Table 3). The shifting of cultivation from autotrophic mode to mixotrophic mode showed increment in the amount of PUFAs from 16.40% to 18.91% while declining the amount of saturated fatty acids (SFAs) from 30.27% to 25.56%. The overall quantities of saturated (SFAs) and monounsaturated fatty acids (MUFAs) were similar in both mixotrophic cultivation modes (with and without sugar source), whereas the PUFAs content increased from 18.91% to 22.12% in mixotrophic cultivation with 2 g/L glucose (GFM; C/N 20) (Table 3). Under nitrogen-limiting conditions (C/N 60), EPA and DHA contents increased further to 18.38% and 3.56%, respectively, as did MUFAs and PUFAs compared to C/N 20 (Table 3). A possible explanation for the high amount of MUFAs in nitrogen-limiting conditions is the elevated synthesis of SFAs in the early stationary phase; these SFAs (stearic acid; C_18:0_) are then converted to MUFAs (oleic acids, C_18:1_) in later stages by Δ9 desaturase, which adds double bonds [38,39]. The addition of extra Δ9 bonds by the specific monooxygenase system requires 1 mole of O_2_ to desaturate one mole of C_18:0_ [40]_._ This adaptation prevents the accumulation of reactive oxygen species under stress conditions [41]. 

The antioxidative property was verified by overexpressing Δ9 fatty acid desaturase in *Cryptococcus curvatus* (CBS 570) [42], whereas the oxidative process was proved using a Δ9 desaturase inhibitor which prevents the conversion of C_18:0_ to C_18:1_ [43]. *P. tricornutum* was cultivated under various conditions of salinity, temperature, nitrogen concentration, and light intensity to evaluate the growth, fatty acid profile, and DHA/EPA ratio [44]. The results were similar to the ones reported here, showing that nitrogen limitation in *P. tricornutum* increased the amount of SFA and MUFAs yet lowered PUFAs [44]. In contrast to our work, Yodsuwan et al. (2017) reported that *P. tricornutum* synthesized more EPA under nitrogen sufficient conditions [30]. The fatty acids profiles that were obtained on BH (C/N, 60) and SH (C/N, 60) were similar to those obtained on GFM at C/N 60 (Table 3). A low DHA/EPA ratio (<0.50) was proposed to have a beneficial effect on mitigating high-fat diet-induced liver damage in mice, whereas a DHA/EPA ratio of 0.50 could alleviate inflammatory risk factors [45]. In the present study, the effect of various modes of cultivations on the DHA/EPA ratio are presented in Table 3. The DHA/EPA ratio increased when cultivation was shifted from photoautotrophic to mixotrophic. The DHA/EPA ratio was highest when *P. tricornutum* was grown in spruce hydrolysate (0.25) as compared to phototrophycally grown cultures (0.12). As DHA and EPA are required to maintain membrane function in microalgae, an increased DHA/EPA ratio might reflect a response to oxidative stress [44]. The DHA/EPA ratio has many regulatory effects on animal metabolisms such as visual, neurological, and the cardiovascular system [45,46], while there are only few reports on the effect of the DHA/EPA ratio in microalage. 

The concentration, yield, and productivity of EPA and DHA by *P. tricornutum* cultivated under photoautotrophic and mixotrophic conditions on various substrates are presented in Table 4. The highest EPA concentration (256.32 mg/L), yield (77.43 mg/g_dry biomass_), and productivity (19.69 mg/L/d) were obtained on spruce hydrolysates and were higher than the corresponding values achieved via mixotrophic cultivation without glucose (79.80 mg/L, 31.66 mg/g_dry biomass_, and 6.14 mg/L/d, respectively) and also with photoautotrophic cultivation (26.86 mg/L, 30.17 mg/g_dry biomass,_ and 2.44 mg/L/d, respectively) (Table 4). Similar results were obtained with DHA concentration (63.08 mg/L), yield (19.05 mg/g_dry biomass_), and productivity (4.85 mg/L/d), all of which were higher than under mixotrophic growth without glucose (9.75 mg/L, 3.86 mg/g_dry biomass_, and 0.75 mg/L/d, respectively) and under photoautotrophic cultivation (3.30 mg/L, 3.70 mg/g_dry biomass_, and 0.30 mg/L/d, respectively). Thus, a switch from photoautotrophic to mixotrophic conditions with glucose had a significant impact on the productivity of EPA and DHA, representing an important step towards the commercial production of EPA and DHA from *P. tricornutum*. In another study, *P. tricornutum* was cultivated mixotrophically on fructose and glycerol in fed-batch and semi-continuous modes. The highest biomass and EPA productivities (1.5 g/L/d and 40 mg/L/d, respectively) were observed in semi-continuous cultures on glycerol [15]. In another work, EPA productivity was 54.8 mg/L/d using the same strain grown photoautotrophically in a 200-L horizontal photobioreactor [36].

Cultivation conditions other than carbon and nitrogen sources have also been seen to affect EPA content in *P. tricornutum*. For example, the addition of light (photosynthetically active radiation) with UV at 8.22 W/m^2^ to photoautotrophically cultivated *P. tricornutum* CS29 increased EPA and DHA from 16.59% and 1.28% to 19.84% and 1.35%, respectively [47]. *P. tricornutum* UTEX 640 cultivated in an airlift tubular photobioreactor outdoors reached a maximum concentration and productivity of EPA of 423 mg/L and 13 mg/L/d, respectively [48]. Hamilton et al. (2016) created a transgenic heterotrophic strain of *P. tricornutum* by co-expressing a ∆5 elongase from *Ostreococcus tauri* and glucose transporter from the moss *Physcomitrella patens* to enhance the accumulation of long-chain omega-3 PUFAs. They reported that the transgenic strains (Pt_Elo5) and (Pt_HElo5_5) those grown in the presence of light and 1% glucose resulted in a lower EPA content (15.9% and 19.0%, respectively) yet a higher DHA content (6.3% and 7.5%, respectively) than the wild-type strain (24.7% and 1.8%) [17]. Moreover, strain Pt_HElo5_5 synthesized little EPA (3.4 µg/mg dry weight) and DHA (1 µg/mg dry weight) when grown heterotrophically on 1% glucose [17]. From these results, we can conclude that mixotrophic cultivation is the most suitable mode of cultivation for the synthesis of high amounts of EPA and DHA, especially when employing wild-type strains. 

### 2.5. Pigment Composition in the Lipids Obtained during Mixotrophic Cultivation of P. tricornutum on Wood Hydrolysates

Besides EPA and DHA, photosynthetic pigments produced by marine microalgae are also of strong interest to researchers for their medicinal or health benefits in antioxidative responses, immunomodulation, retinal degradation, muscular dystrophy, and cancer prevention [49]. *P. tricornutum* is a known producer of fucoxanthin which induces G1 cell-cycle arrest and can be utilized to treat cancer [49]. However, the presence of chlorophyll, carotenoids, tocopherols, and phospholipids in edible oils has been shown to increase their shelf-life when stored in the dark and prevent them from autooxidation due to antioxidant and prooxidant activity [50].

**Figure 3:**
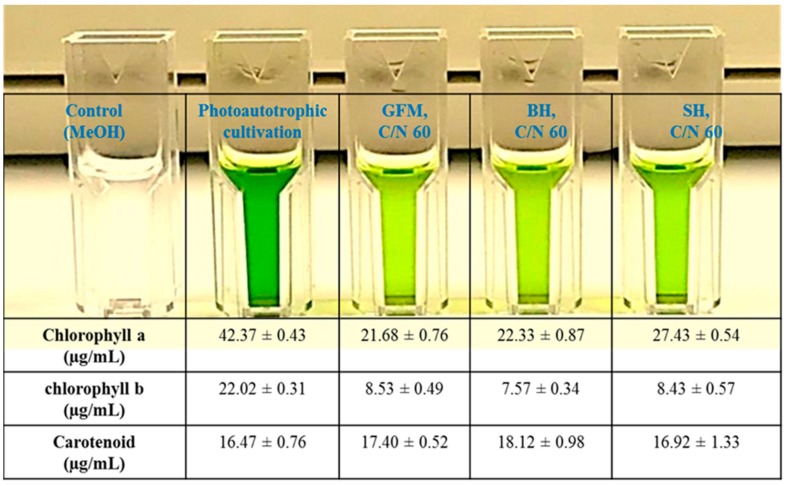
Estimation of chlorophyll a, b, and carotenoids in *P. tricornutum* grown under mixotrophic cultivation for 312 h on GFM, BH, and SH, (C/N 60) and a comparison with photoautotrophic cultivation.

A high ratio of carotenoid to total chlorophyll, including a high ratio of *chl* a to *chl* b, in microalgae grown under nitrogen starvation conditions is responsible for a reduction in light-harvesting complexes and the accumulation of lipids to protect cells against oxidative stress [51]. *Chl* a, *chl* b, and carotenoids were determined in *P. tricornutum* grown photoautotrophically and mixotrophically on GFM, BH, and SH (Figure 3). *P. tricornutum* cultivated under photoautotrophic conditions had the highest amount of *chl* a (42.37 µg/mL) and *chl* b (22.02 µg/mL), with the corresponding values in mixotrophically grown algae being considerably lower (Figure 3). In contrast, the amount of carotenoids was similar under both autotrophic and mixotrophic conditions, ranging from 16.47 µg/mL to 18.12 µg/mL (Figure 3).

*Chl* a, *chl* c, and carotenoids are the main photosynthetic pigments, with *chl* a in particular acting as a reaction center for the light harvesting complex of *P. tricornutum* [52]. Similarly to our present results, previous photoautotrophic cultivation of *P. tricornutum* yielded higher amounts of *chl* a and *chl* b than mixotrophic cultivations with glucose, glycerol, and acetate [19]. Glucose has been reported to lower the evolution of photosynthetic O_2_ as a result of reduced activity of ribulose-1,5-bisphosphate carboxylase/oxygenase (Rubisco) as well as photosystem II reaction centre protein D1 in *Galdieria sulphuraria* [53]. Moreover, limited nitrogen availability has been shown to reduce the synthesis of photosynthetic pigments and proteins in *P. tricornutum* CCMP 2561 [54] and similar results were observed in our study during mixotrophic cultivation of *P. tricornutum* under the nitrogen-limited condition (see Figure 3). 

## 3. Materials and Methods

### 3.1. Materials

*P. tricornutum* SAG 1090-6 was procured from the Culture Collection of Algae (international acronym SAG) at Göttingen University, Germany. All the chemicals, including cultivation media and Guillard’s (F/2) marine water enrichment solution (G0154), were purchased from Sigma Aldrich (St. Louis, MO, USA). Artificial seawater was prepared using reed aquarium salts (Tetra marine sea salt, Melle, Germany) in distilled water. Yeast extract (total N 11.6% and amino N 6.2%) used as an organic nitrogen source (Cat. No. 92144) was procured from Sigma, Aldrich, (St. Louis, MO, USA). All solvents and reagents were of HPLC grade. 

### 3.2. Medium and Culture Conditions

The green microalga *P. tricornutum* was maintained at 16 °C on F/2 agar plates containing tetracycline (20 µg/mL) to eliminate bacterial contamination. Once new colonies appeared on the plate, they were streaked on new agar plates without antibiotic. Initially, the microalga was grown photoautotrophically in a photobioreactor (Multi-Cultivator MC 1000-OD; Photon Systems Instruments, Drasov, Czech Republic) on F/2 marine water enrichment solution and sodium nitrate as inorganic nitrogen source under 14/10-h light/dark regimen (intensity of 100 μmol/m^2^/s^1^) at 20 ± 1 °C. Salinity and pH were adjusted to 28 ppt and 8.0 prior to sterilization, respectively, and aeration was provided by air bubbling at atmospheric pressure. The F/2 marine water enrichment solution had the following composition (mg per liter): NaNO_3_, 75; NaH_2_PO_4_, 4.411; FeCl_3_. 6H_2_O, 3.15; CuSO_4_.5H_2_O, 0.01; ZnSO_4_.7H_2_O, 0.022; MnCl_2_.4H_2_O, 0.18; Na_2_MoO_4_.2H_2_O, 0.006; CoCl_2_.6H_2_O, 0.01; biotin, 0.005; EDTA disodium.2H_2_O, 4.36; thiamine.HCl, 0.1; vitamin B12, 0.005 mg. The culture conditions that were used in this study, such as light intensity, salinity, temperature, and pH, were optimized earlier for the maximum biomass and lipid accumulation by Qiao et al. (2016) [44]. 

### 3.3. Optimization of Biomass and Lipid Production under Mixotrophic Cultivation

For mixotrophic cultivation, cells were harvested from the photoautotrophically grown cultures by centrifugation at 3000× *g* for 10 min under aseptic conditions. The algal pellet was washed with 0.9% saline (NaCl) to remove residual medium and cell density was adjusted to 6.9–9.2 × 10^8^ cells/mL using 0.9% saline. All mixotrophic cultivations were carried out in a photobioreactor (see Section 3.2) consisting of eight test tubes of 85 mL capacity. Cultivation conditions were akin to the photoautotrophic ones except for the addition of glucose as a carbon source to F/2 medium. To examine the effect of initial glucose on biomass and lipid accumulation under the mixotrophic mode, five different glucose concentrations, ranging from 2 g/L to 10 g/L, were added at 2 g/L steps to basal F/2 medium (designated as glucose F/2 medium, GFM). The C/N ratio was adjusted to 20 by adding an appropriate amount of yeast extract on the basis of total N present in yeast extract (11.6% of total nitrogen with 6.2% amino N) and total C of the sugar source. One experiment was also performed without the addition of any sugar source in yeast extract-based F/2 medium and was considered the control experiment for mixotrophic cultivation without sugar source (glucose). After optimization of biomass production under different glucose concentrations, lipid production was optimized at varying C/N ratios (20, 40, 60, 80, and 100) by adjusting the amount of yeast extract. 

### 3.4. Batch Cultivations of P. tricornutum Using Hydrolysates from Organosolv-Pretreated Birch and Spruce Woodchips

Birch and spruce woodchips (milled to <1 mm) were pretreated using a hybrid organosolv–steam explosion method, as described previously [21,22]. More specifically, birch was pretreated at 200 °C for 15 min with 60% *v*/*v* ethanol and 1% w/w_biomass_ H_2_SO_4_ for 15 min, whereas spruce was pretreated at 200 °C for 30 min with 52% *v*/*v* ethanol and 1% w/w_biomass_ H_2_SO_4_. At the end of pretreatment, the solids were separated from the liquor, washed with ethanol, and air-dried. Enzymatic hydrolysis of pretreated solids was carried out as described previously [23]. Briefly, 100 g of a 10% *w*/*w* solids solution in 50 mM citrate-phosphate buffer (pH 5) was prepared in 500 mL Erlenmeyer flasks. The commercial Cellic CTec2 solution (Novozymes A/S, Bagsværd, Denmark) was applied at an enzyme load of 20 FPU/g_solids_ and hydrolysis was performed for 48 h at 50 °C and 180 rpm. At the end of hydrolysis, the liquid was separated from the solids by centrifugation and glucose concentration was determined by HPLC [23]. Subsequently, birch (BH) and spruce (SH) hydrolysates were mixed with basal F/2 medium to achieve the glucose concentration known to be optimal for maximum biomass and lipid yield. Yeast extract was used to achieve the desired C/N ratio. Batch mixotrophic cultivations were carried out in a 1.9-L flat-panel airlift-photobioreactor (Labfors 5, Infors AG, Basel, Switzerland). Light intensity was adjusted to 100 µmol/m^2^/s^1^ by an LED panel (adjustable 260 water-cooled high-power LEDs) under a 14/10-h light/dark regimen. The other culture conditions such as pH and salinity of medium were adjusted to 8.0 and 28 ppt while the temperature was adjusted to 20 ± 1 °C. Sterile air was sparged at atmospheric pressure throughout the experiments. Samples from the photobioreactor were taken on a regular basis to determine cell density, cell dry weight, lipid content, and residual glucose in the medium.

### 3.5. Analytical Methods

During cultivation in the Multi-Cultivator MC 1000-OD, cell growth was monitored every 10 min by in situ density measurements at 680 nm and 730 nm. Cell dry weight (g/L) was determined gravimetrically after centrifuging 10 mL of culture broth at 4000× *g* for 10 min. The cell pellet was washed twice with distilled water to remove residual medium and dried in an oven at 55 °C until a constant weight was attained. Biomass productivity was calculated by the following equation:

(1) B=(W2−W1)(T2−T1)

where *B* is biomass productivity (g/L/day) and *W*1 and *W*2 are cell dry weights (g/L) on days *T*1 (start point of cultivation) and *T*2 (endpoint of cultivation), respectively.

The lipids were extracted from the dried biomass according to the protocol described in Patel et al., 2018. Briefly, the dried biomass from 25 mL of culture broth was mechanically crushed using a mortar and pestle to form a fine powder, followed by extraction with a solvent mixture (chloroform:methanol, 2:1 *v*/*v*) overnight at room temperature, with constant shaking. The slurry was filtered using a 0.22-μm filter and the solvent containing the lipids was transferred to pre-weighed glass vials. The glass vials were dried under vacuum and weighed to estimate the total lipid concentration (g/L). 

Lipid content (% *w*/*w*) on the basis of cell dry weight (g/L) was calculated by the following equation:

(2) L=TLCCDW

where *L* is lipid content (% *w*/*w*) and *TLC* and *CDW* are total lipid concentration (g/L) and cell dry weight concentration (g/L), respectively.

Lipid productivity (*P*), expressed in mg/L/day, was calculated by the following equation:

(3) P=L×B100

Residual glucose from the mixotrophic cultivations was measured by HPLC (PerkinElmer, Waltham, MA, USA) equipped with a refractive index detector and a Bio-Rad Aminex HPX-87N column (BioRad, Hercules, CA, USA). The column was maintained at 85 °C and 0.01 M Na_2_HPO_4_ was used as the mobile phase at a flow rate of 0.6 mL/min. Sugar consumption was calculated with the following equation:

(4) $$S=\frac{Gt1-Gt2}{Gt1}\times 100$$

where *S* is the % sugar consumption, *Gt*1 is the amount of initial sugar added (g/L), and *Gt*2 is the residual sugar left at each sampling time.

Photosynthetic pigments (*chlorophyll a*, *chlorophyll b*, and *carotenoids*) were analyzed by harvesting 2 mL of culture (wet biomass) followed by the addition of methanol (2 mL) and incubation at 45 °C for 24 h. Cell debris were removed by centrifugation and the supernatant was used to measure absorbance at 665.2 nm, 652.4 nm, and 470 nm with a UV/visible spectrophotometer. The amounts of pigment were determined using the following equations:

*Chlorophyll a* (*Chl a*; µg/mL) = 16.72 *A*_665.2_ − 9.16 *A*_652.4_

*Chlorophyll b* (*Chl b*; µg/mL) = 34.09 *A*_652.4_ − 15.28 *A*_665.2_

*Carotenoids* (µg/mL) = 1000 *A*_470_ − (1.63 *Chl a* − 104.9 *Chl b*)/221

where *A* is absorbance at a particular wavelength [55].

The lipid profile of this alga grown on various substrates was estimated on the basis of its transesterified products by GC-FID. Total extracted lipids were transesterified with an acid catalyst (8 mL of 6% methanolic H_2_SO_4_), as described previously [23]. The obtained fatty acid methyl esters (FAMEs) were analyzed by GC-FID (Agilent, Santa Clara, CA, USA) using a capillary column (Select FAME; dimensions 50 m × 0.25 mm ID and 0.25 μm film thickness) under previously reported operating conditions [23].

### 3.6. Statistical Analysis

Data are expressed as means ± standard deviation of three independent recorded values as all experiments were repeated three times. One-way analysis of variance (ANOVA) using Microsoft Office Excel 2016 (Microsoft, Redmond, WA, USA) with *p* < 0.05 was used for data acceptance.

## 4. Conclusions

The present study shows that mixotrophic growth of *P. tricornutum* led to higher EPA and DHA productivity than photoautotrophic growth. The use of nitrogen-limiting conditions further increased EPA and DHA content in the obtained lipids. Birch and spruce hydrolysates can serve as an excellent source of glucose for *P. tricornutum*, enabling approximately 3.11- and 3.2-times higher EPA productivity, respectively, than during photoautotrophic cultivation. To the best of our knowledge, this is the first report where forest biomass is used for microalgae growth and production of nutraceutical lipids. Accordingly, forest biomass can serve as a novel low-cost renewable resource for microalgae PUFA production.

## Figures and Tables

**Table 1:** Comparison of photoautotrophic and mixotrophic cultivations and the effect of different initial concentrations of glucose on cell dry weight (g/L), total lipid concentration (g/L), lipid content (% *w*/*w*), biomass yield (g/g_substrate_), lipid yield (g/g_substrate_), and residual glucose (g/L) of *P. tricornutum* after 312 h of culture.

Initial Glucose Concentration (g/L) in GFM	Cell Dry Weight (g/L)	Biomass Productivity ^#^ (g/L/d)	Lipid Concentration (g/L)	Lipid Content (%, *w*/*w*)	Lipid Productivity ^#^ (g/L/d)	Biomass Yield (g/g_substrate_)	Lipid Yield (g/g_substrate_)	Residual Glucose Concentration (g/L)
Photoautotrophic cultivation	0.89 ± 0.11	0.081± 0.001	0.20 ± 0.06	22.47 ± 0.23	0.018 ± 0.001	-	-	-
0 (control)	2.52 ± 0.14	0.193 ± 0.002	0.57 ± 0.09	22.62 ± 0.28	0.043 ± 0.007	-	-	-
2	3.38 ± 0.16	0.260 ± 0.003	0.88 ± 0.11	26.03 ± 0.45	0.067 ± 0.009	1.69 ± 0.19	0.44 ± 0.09	0.00 ± 0.00
4	4.10 ± 0.21	0.315 ± 0.005	1.08 ± 0.12	26.34 ± 0.21	0.083 ± 0.001	1.31 ± 0.21	0.34 ± 0.08	0.86 ± 0.17
6	4.14 ± 0.31	0.318 ± 0.008	1.12 ± 0.21	27.05 ± 0.71	0.086 ± 0.002	1.29 ± 0.13	0.35 ± 0.07	2.80 ± 0.23
8	4.24 ± 0.19	0.326 ± 0.004	1.15 ± 0.17	27.12 ± 0.87	0.088 ± 0.001	1.19 ± 0.21	0.32 ± 0.04	4.45 ± 0.41
10	4.32 ± 0.32	0.332 ± 0.009	1.16 ± 0.23	26.85 ± 0.76	0.089 ± 0.002	1.34 ± 0.19	0.36 ± 0.09	6.78 ± 0.37

*^#^* The total biomass productivity and lipid productivity were calculated when the cell dry weight reached its highest value.

**Table 2:** Quantitative estimation of cell dry weight, total lipid concentration, lipid content, biomass productivity, and lipid productivity of *P. tricornutum* cultivated under photoautotrophic and mixotrophic mode on various substrates.

Parameters	Photoautotrophic Cultivation	GFM (C/N, 60)	BH (C/N, 60)	SH (C/N, 60)
Cell dry weight (g/L)	0.89 ± 0.11	3.15 ± 0.53	3.23 ± 0.32	3.31 ± 0.28
Biomass Productivity ^#^ (g/L/d)	0.081± 0.001	0.242 ± 0.005	0.248 ± 0.004	0.254 ± 0.007
Lipids concentration (g/L)	0.20 ± 0.06	1.21 ± 0.19	1.26 ± 0.11	1.29 ± 0.18
Lipid content (%, *w*/*w*)	22.47 ± 0.23	38.41 ± 0.21	39.00 ± 0.23	38.97 ± 0.43
Lipids productivity ^#^ (mg/L/d)	18.18 ± 0.34	93.07 ± 0.68	97.00 ± 0.85	99.23 ± 1.09

*^#^* The total biomass productivity and lipid productivity were calculated when the cell dry weight reached its highest value.

**Table 3:** Analysis of fatty acids profile of *P. tricornutum* cultivated under photoautotrophic and mixotrophic mode on various substrates.

Fatty Acids (%) in Total Lipid		Photoautotrophic Cultivation		Mixotrophic Cultivation Without Glucose		GFM; C/N 20		GFM; C/N 60		BH; C/N 60		SH; C/N 60	
**Saturated Fatty Acids (SFAs)**	(C_14:0_)	8.24	**30.27**	7.18	**25.56**	2.60	**24.62**	2.9	**26.33**	3.1	**27.1**	2.65	**26.13**
	(C_16:0_)	15.39		13.62		12.53		11.01		13.23		12.32	
	(C_18:0_)	4.32		2.96		1.72		3.8		2.89		2.65	
	(C_20:0_)	2.32		1.80		3.54		3.65		3.43		3.87	
	(C_24:0_)	-		-		4.23		4.97		4.45		4.64	
**Mono Unsaturated Fatty Acids (MUFAs)**	(C_16:1_)	17.23	**34.56**	13.62	**34.23**	15.99	**34.48**	17.17	**38.78**	17.65	**39.12**	17.87	**39.61**
	(C_18:1_ n9t)	15.21		18.3		15.37		16.96		16.34		16.87	
	(C_18:1_ n9c)	2.12		2.31		3.12		4.65		5.13		4.87	
**Poly Unsaturated Fatty Acids (PUFAs)**	(C_18:2_ n6c)	1.32	**16.40**	3.20	**18.91**	2.54	**22.12**	2.8	**24.74**	2.56	**26.68**	2.71	**27.47**
	(C_18:3_ n3)	-		-		-		-		-		-	
	(C_20:5_ n3) EPA	13.43		14.0		16.76		18.38		19.80		19.87	
	(C_22:6_ n3) DHA	1.65		1.71		2.82		3.56		4.32		4.89	
**DHA/EPA**		0.12		0.12		0.17		0.19		0.22		0.25	
**Total fatty acids**		81.23		78.70		81.22		89.85		92.9		93.21	

**Table 4:** EPA and DHA concentration (mg/L) and productivity (mg/L/d) by *P. tricornutum* cultivated under photoautotrophic and mixotrophic cultivation mode on various substrates.

Parameters	Photoautotrophic Mode of Cultivation	Mixotrophic Mode of Cultivation				
		Without Glucose	GSM (Glucose, 2g/L) C/N;20	GSM (Glucose, 2g/L) C/N;60	BH (Glucose, 2g/L) C/N;60	SH (Glucose, 2g/L) C/N;60
**Total EPA concentration (mg/L)**	26.86	79.80	147.48	222.39	249.48	256.32
**EPA yield (mg/g_dry biomass_)**	30.17	31.66	43.63	70.60	77.23	77.43
**EPA productivity (mg/L/d)**	2.44	6.14	11.31	17.07	19.15	19.69
**Total DHA concentration (mg/L)**	3.30	9.75	24.82	43.08	54.43	63.08
**DHA yield (mg/g_dry biomass_)**	3.70	3.86	7.34	13.67	16.85	19.05
**DHA productivity (mg/L/d)**	0.30	0.75	1.91	3.32	4.18	4.85
